# Energy Saving in Office Buildings: Are Feedback and Commitment-Making Useful Instruments to Trigger Change?

**DOI:** 10.1007/s10745-015-9783-8

**Published:** 2015-09-29

**Authors:** Anne Marike Lokhorst, Henk Staats, Jochem van Iterson

**Affiliations:** Wageningen University, Wageningen, The Netherlands; Leiden University, Leiden, The Netherlands

**Keywords:** Energy saving, Interventions, Feedback, Commitment-making, Organizations, Leiden

## Abstract

This study focuses on energy saving in an office environment. We developed and tested an intervention that contained both the administration of feedback as well as commitment-making: two techniques that are often described in the literature as successful, especially when combined. Using a sample of 146 employees, we tested the intervention’s effectiveness for our sample in terms of behavior change. Our results show some effects, but these were irrespective of experimental category. We use this failed experiment to reflect upon critical aspects of the design and implementation of intervention, and provide ideas on how such interventions can be improved.

## Introduction

One of the challenges currently facing company managers is how to reduce the energy use of their organizations. It seems clear that decreasing energy use is beneficial both for the environment and for the company’s energy expenditures. However, merely informing employees of these benefits and requesting that they reduce their use of light, heat, and cooling does not appear to necessarily lead to changed behaviors (Kollmuss and Agyeman 2002). Effecting change towards more environmentally sustainable behavior is complicated.

Acknowledging this, a substantial body of psychological research has focused on developing and testing theory-based behavioral change techniques (e.g., Abrahamse *et al.*^2007^). Such techniques appear to be promising, but their success is contingent on certain boundary conditions that are not always clear.

Energy consumption is a textbook example of a domain where changes in behavior have great beneficial effects on greenhouse gas emissions and the accompanying burdens of climate change. It is therefore not surprising that this set of behaviors that has attracted particular attention from social scientists over the recent years (e.g., De Vries *et al.*^2011^; Allcott and Mullainathan 2010; Abrahamse *et al.*^2007^, ^2005^). Such research has to some extent focused on understanding the determinants of energy use (Mills and Schleich 2012; Abrahamse and Steg 2009), and more particularly on developing and testing interventions to change different energy related behaviors (e.g., Abrahamse *et al.*^2007^, ^2005^; Staats *et al.*^2004^).

For the most part, these studies have concentrated on household or residential energy use. Few studies have explicitly focused on changing energy use in public or private organizations (Paillé and Boiral 2013; Stern 2011), although Matthies and Hansmeier (2008) discuss an intervention at a German university, which combined information, commitment and prompts. This intervention was successful: participants showed significant behavior change and heating energy went down by 6 % during the intervention period. In addition, Carrico and Riemer (2011) evaluated the effect of group-level feedback and peer education in a sample of university employees and conclude that both resulted in significant energy reductions. Staats *et al.* (2000) investigated whether several informational strategies – among them, brochures and different types of feedback – were effective in promoting energy saving in an office building. They report improvements directly after the intervention, with partial behavior maintenance 1 year later. Finally, Chen *et al.* (2012) describe a study in which junior researchers at a university received feedback on their energy use in the form of a digital pet on their computer screens. This intervention was successful in realizing conservation objectives.

Energy saving in an office environment has several characteristics that set it apart from household energy saving. For example, household energy saving presents a clear financial incentive in reduced energy bills. For employees working in office environments, however, there are no such individual financial incentives. Second, since organization employees are usually more numerous than household members, incentives to energy saving in the office are not so readily apparent and are thus less efficacious. Even when information about energy use is communicated, it is usually presented at the group level, reducing employees’ sense of individual responsibility. These constraints make energy saving in an office environment more challenging than household energy saving and should be taken into account when designing interventions.

Two categories of energy related behaviors can be distinguished: curtailment and increased efficiency (see Gardner and Stern 2002). While efficiency behaviors refer to one-time decision making, such as installing solar panels, curtailment behaviors are actions that need to occur on a frequent basis in order to be effective, e.g., switching off lights and electrical appliances. In organizational settings, decisions concerning increased efficiency will likely be made by (higher) management. Curtailment behaviors, however, are possible for all personnel and afford substantial energy savings. In the current study, we focus on testing interventions to improve curtailment behaviors.

Over the last few years there has been increased interest in commitment-making as a tool for promoting change in curtailment behaviors (Lokhorst *et al.*^2013^). This technique requires individuals to make a formal pledge to change aspects of their behavior, usually related to specified goals. Several studies (e.g., Matthies *et al.*^2006^; Wang and Katzev 1990) have shown that people seem inclined to adhere to their commitments and thus exhibit behavior change, and these findings have been acknowledged in multiple reviews (Osbaldiston and Schott 2012; Abrahamse *et al.*^2005^; DeYoung 1993; Dwyer *et al.*^1993^). Indeed, a meta-analysis by Lokhorst *et al.* (2013) showed that commitment-making is effective across different environmental behaviors, especially when combined with other strategies. These studies also provide some ideas about possible underlying mechanisms accounting for the effect that commitment has on behavior. Specifically, they propose three (related) possible mediators. First, making a commitment might change people’s ideas about what they value; if they believe they have freely chosen to commit to changing their behavior in relation to a specified goal, e.g., energy conservation, then that must mean that the goal, e.g., mitigation of climate warming, is important to them. Since people are socialized to be consistent (Cialdini 2001) they will consequently be motivated to adjust their behavior to reflect the value they place on the goal. Second, the studies propose making a commitment sets in motion a process of cognitive elaboration that results in self-persuasion. Again, it is important that people believe they make their commitment voluntarily, as they will then be motivated to adjust their behavior to be consistent with their valuation of the goal and ultimately transform their short-term commitment into long-term self-directed behavior; that is, they will persuade themselves that the commitment and consequent adjusted behavior are worthwhile. Finally, there is the possibility of a normative mechanism, whereby commitments made in public lead to adherence because of negative social sanctions that might follow from reneging on them.

These potential mediators overlap to a certain extent, and it is very possible that they are mutually supportive. And while Lokhorst *et al.* (2013) provide some evidence for all three, they have to the best of our knowledge never been relatively assessed in the context of commitment-making.

As noted above, the combination of commitment-making with other strategies appeared most successful in modifying behaviors - especially with the administration of feedback providing people with information about the way they are currently performing a behavior, have performed a behavior earlier, or about the outcomes of their behavior (Osbaldiston and Schott 2012). In the case of energy conservation, this would typically involve showing people their current and/or past energy use in the form of meter readings or energy bills. Reviews on the effectiveness of feedback have shown that it is most effective when it is immediate (Darby 2006), provided frequently (Fischer 2008; Abrahamse *et al.*^2005^), and when a relevant comparison is made, for instance with earlier behavior or with that of others. Allcott (2011) analyzed a series of feedback programs aimed at the reduction of energy use and estimated that the average program reduces energy consumption by 2.0 %. Due to the scale of the programs and the longevity of the changes reported this amounts to an impressive quantity of energy saved.

The combination of commitment-making and feedback can create an especially successful track to behavioral change (DeLeon and Fuqua 1995). The making of a commitment can signal to the individual that this is a topic they value about and find important. Receiving feedback on their behavior can serve as a prompt and reminder of their commitment, and this may set in motion a process of cognitive elaboration which enables them to develop a strong and accessible attitude that in turn serves to both remind and motivate them to engage in the appropriate behavior (see also Lokhorst *et al.*^2013^).

Since most of these studies were not carried out in office contexts, we designed this study to test the effectiveness of commitment-making and feedback for reducing energy use in an organizational setting. In light of previous studies, we expected both commitment-making and feedback to be effective in comparison to a control group, especially when the two are combined. Specifically, we expected that the making of a commitment to save energy (*Hypothesis 1*) as well as receiving feedback on energy use (*Hypothesis 2*) would lead to changes in behavior as well as environmental concern compared to a control group. Also, we predicted that the combination of feedback plus commitment would be more effective than feedback or commitment alone (*Hypothesis 3*).

## Methods

### Participants and Design

The participants in this study were all 146 administrative employees (thus excluding maintenance and cafeteria personnel) of the municipality of Leiden who have a permanent office in the City Hall of Leiden, a city of 120,000 inhabitants in the Netherlands. The study ran from the second week of November until the end of the third week of December, 2010. Participants were divided into four categories: Commitment and Feedback (CF), Commitment Only (CO), Feedback Only (FO), and Control (Ctrl). Participants in the CF category agreed to commit themselves to saving energy for the next 6 weeks and receive 3-weekly feedback on the self-reported behavior of their group. CO participants agreed to commit themselves to saving energy, but did not receive any feedback. FO participants received a weekly feedback e-mail based on their energy consumption of electricity (for lights, computers and other electronic equipment) and heating, and were also provided with a digital photo-frame that showed feedback updated every hour. Ctrl participants were asked to fill out the first and last questionnaire, but did not receive any feedback. Participants were divided into the four categories based on the floors they worked on to avoid carry-over effects across conditions. The only exception was the FO category (*N* = 25; 17 %) for which meters to measure energy consumption were installed in 12 offices on the first, second, third and fourth floors. Participants on the ground and first floors were assigned to the CF categories (*N* = 53; 36 %); participants on the second and third floor were assigned to the Ctrl category (*N* = 29; 20 %); and participants on the fourth and fifth floor were assigned to the CO category (*N* = 39; 26 %). The floors serve different functions: the ground floor includes service areas for the public, the second floor has the large boardroom where the city council convenes its meetings, and the fifth floor has less space; thus the number of offices per floor differs.

### Survey: Measures and Implementation

Several employees at the City Hall, Leiden, who were subsequently excluded from the main study, participated in a pilot questionnaire for which no problems were reported. All participants were asked to fill out a pre-test questionnaire that included some demographic questions (age, gender, working hours, number of persons per office). We measured nine self-reported behaviors, such as turning off the light when leaving the office, lowering the thermostat, etc. The response scales were formulated so that higher scores indicated more energy use (see Table 1 for pre- and post-intervention scores of the nine behaviors). Information about the study was provided according to category

**Table 1:** Behavior items: Means and standard deviations both pre- and post-intervention

Items	Answering scales	Pre-intervention	Post-intervention
Behavior measures			
During the last 2 weeks I would switch off the lights when I would leave my office for…	1. 0–30 min 2. 30–60 min 3. 1–2 h 4. 2–4 h 5. More than 4 h 6. When I went home 7. Never	M = 4.86, SD = 1.88	M = 4.39, SD = 2.06
During the last 2 weeks I would switch off my ceiling lights when there was sufficient natural light	1. Always 2. Often 3. Sometimes 4. Seldom 5. Never	M = 2.94, SD = 1.60	M = 3.13, SD = 1.57
During the last 2 weeks I would switch my computer to standby mode when I wasn’t using it for…	1. 0–30 min 2. 30–60 min 3. 1–2 h 4. 2–4 h 5. More than 4 h 6. When I went home 7. Never	M = 4.84, SD = 2.34	M = 4.91, SD = 2.35
During the last 2 weeks I would switch my computer to sleep mode when I wasn’t using it for…	1. 0–30 min 2. 30–60 min 3. 1–2 h 4. 2–4 h 5. More than 4 h 6. When I went home 7. Never	M = 6.09, SD = 1.93	M = 5.61, SD = 2.01
During the last 2 weeks I would switch off my computer when I wasn’t using it for…	1. 0–30 min 2. 30–60 min 3. 1–2 h 4. 2–4 h 5. More than 4 h 6. When I went home Never	M = 5.95, SD = 0.81	M = 5.81, SD = 0.88
During the last 2 weeks I kept my office thermostat (usually) at …	1. Off 2. 1 2. 2 3. 3 4. 4 5. 5 (max)	M = 2.33, SD = 1.43	M = 2.31, SD = 1.33
During the last 2 weeks I would lower my thermostat when I found it too hot in my office	1. Always 2. Often 3. Sometimes 4. Seldom 5. Never	M = 3.35, SD = 2.07	M = 2.93, SD = 2.02
During the last 2 weeks when central heating was on, the door was closed	1. Always 2. Often 3. Sometimes 4. Seldom 5. Never	M = 3.68, SD = 1.41	M = 3.34, SD = 1.44
During the last 2 weeks when central heating was on, the windows were closed	1. Always 2. Often 3. Sometimes 4. Seldom 5. Never	M = 1.53, SD = 1.00	M = 1.40, SD = 0.93

.

The post-test questionnaire included the same behavior questions as well as five items to measure environmental concern. Four of these were chosen to reflect the variables of the Theory of Planned Behavior (Ajzen 1991): attitude, subjective norm, perceived behavioral control, and intention. We used single-item scales for each of the concepts to keep the questionnaire as short as possible . We added an item to measure personal norm, an important determinant of energy behavior (Harland *et al.*^1999^), e.g., ‘I find it easy to conserve energy in my office,’ measuring perceived behavioral control, and ‘I feel guilty when I do not conserve energy in my office,’ measuring personal norm. Participants rated their answers on 5-points Likert scales that ranged from ‘completely agree’ to ‘completely disagree’. Reliability of the environmental concern scale was good (Cronbach alpha = .73).

### Implementation

All participants received a brochure after collecting the pre-test questionnaire, containing tips for saving energy. It explained how to use the stand-by and sleep function on the computer and the amount of energy that could be saved by doing so. It also gave a brief explanation of the heating system in City Hall and some general tips on efficient use of electronic equipment.

Participants in the commitment categories (CF and CO) received a request at pre-test to commit themselves for the next 6 weeks to consume as little energy as possible in their offices. They were asked to write “Yes” or “No” on the form with this request and also to write down their names and e-mail addresses. It was indicated on the form that a list of the names of the people willing to participate would be e-mailed to all employees, making this both a written and public commitment. It was also stated that the results of all participants (as a group) would be e-mailed to all employees at the conclusion of the study.

**Fig. 1:**
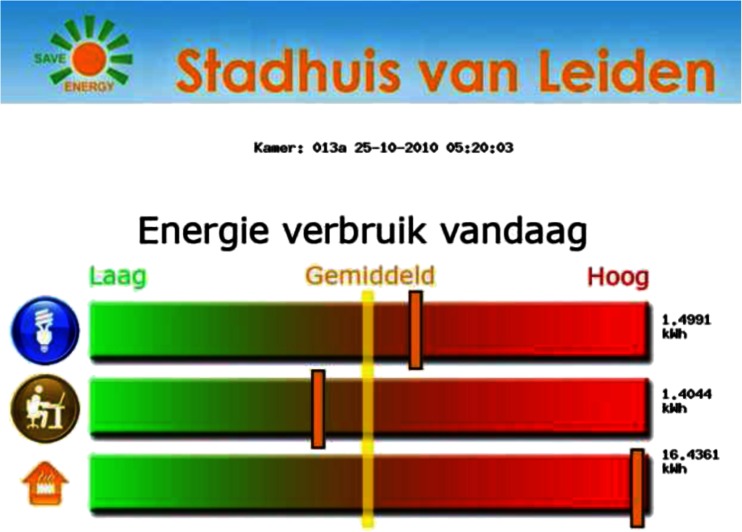
Feedback presentation in the Feedback only condition. The logos represent energy use in 3 different categories: lights, computers, and thermostat. The uninterrupted (*yellow*) line in the middle of the three horizontal bars indicates average use over 3 weeks, and the short (*orange*) lines in each bar show the use per room on a specific time and day

Participants in the FO category received weekly feedback e-mails containing information on their energy consumption of the last week compared to their average consumption in the last 4 weeks (percentage increased or decreased consumption). Three bar charts represented daily energy consumption separately for the desk and for the ceiling lights (in kWh), and a line chart represented the energy consumption over the weeks (with separate lines for desk and light consumption). Total and average consumption were given in kWh. In addition, participants received hourly updated pictures on digital photo frames - one was provided for each office in this category – containing information on energy consumption of desks (collectively for the entire office as opposed to personal energy consumption in the feedback e-mails), ceiling lights and heating. Consumption in these three categories was compared to a 3 weekly average of that specific day and time, and was listed in three separate bars, one for each device, and presented by colors ranging from green (left, ‘below average’) to red (right, ‘above average’) with average consumption in the middle (Fig. 1).

Participants in the CF category received feedback on their baseline scores of self-reported data on energy consumption and again regarding the following 3 weeks. This feedback was communicated in two flyers: one after the pre-test and one after the mid-term test containing bar charts of the collective answers in this category with some explanatory text. The second flyer compared the answers given on the pre-test and mid-term test.

### Analyses

To test our hypotheses we performed a series of repeated ANOVA with time (pre- versus post-test) and experimental category as the independent variables and our nine energy behaviors as the dependent variables. Such an analysis allows testing for differences in energy behaviors in time, and whether or not such differences are caused by our experimental design. To explore the significance of the immediate social context of the behavior we included the number of persons per office as a covariate. We performed a similar analysis on the environmental concern measure. We chose hierarchical decomposition of the sum-of-squares as the model, which we considered most appropriate for this field experiment with an unbalanced design (in which participants per cell of the design are not equal; Stevens 1990: 119–120). This implies that effects due to time are calculated first and effects of the different experimental conditions second. This is a conservative approach that we deemed the most appropriate for this study.

## Results

### Participants and Responses

Of the participants in this study 57.4 % were female. Ages ranged from 20 to 64 years old (*M* = 44.64, *SD* = 10.57), and the number of persons per office from 1 to 8 (*M* = 3.1, *SD* = 1.45).

At pre-test, there were a total of 146 participants. All received the first questionnaire. We received 130 filled-out questionnaires. Of the 92 participants (initially assigned to one of the two commitment categories) we asked to commit themselves, 28 complied. Participants who refused to commit remained included in the commitment categories. The second questionnaire was distributed to 54 participants in the CF category, of which 32 were returned completed. The third questionnaire was distributed to 130 participants, and 108 were returned completed (Table 3 in the Appendix).

### Behavior Change

For switching off the lights (when alone in the office) we found an effect of time, *F*(1,84) = 7.38, *p* < .01, η_p_^2^ = .08, marginally qualified by an interaction effect of time by persons per office, *F*(1, 84) = 3.14, *p* = .08, η_p_^2^ = .04 (Table 1). Generally, participants improved their behavior somewhat. The effects demonstrate that the improvement is not dependent on the experimental category but varies with the number of persons per office. Closer inspection shows that effects are positive, i.e., lower energy use, in the offices with four persons or fewer, and slightly negative in the offices occupied by five or more persons. For switching off part of the ceiling lights when enough light entered the office from outside there were no effects at all.

For switching the computer to standby mode we found an effect of time, *F* (1, 85) = 5.17, *p* < .05, η_p_^2^ = .06, but no effects of category, number of persons per office or their interactions. Participants generally switched to standby mode when not using the computer for shorter periods after the intervention period.

For switching the computer to sleep mode, we found a marginal effect of time, *F* (1,82) = 2.91, *p* < .10, η_p_^2^ = .03, such that during the course of our study, participants showed an overall positive effect: they were quicker to switch their computer to sleep mode (*M* = 6.0 before intervention versus *M* = 5.6 after intervention). This effect cannot, however, be attributed to specific conditions in our intervention as none of the effects for category, number of persons per office or any of the interactions were significant.

Entirely switching off the computer did not change at all during the course of the intervention. None of the effects related to the intervention was significant although the pattern of behavior apparently was somewhat different depending on the number of persons per office, *F*(1, 86) = 6.42, *p* < .02,, η_p_^2^ = .07, suggesting that in the offices with five or more people participants switched off their computer for shorter periods than in the offices with fewer persons.

For keeping the office thermostat at a certain level, we found an interaction effect of time*condition, *F*(3, 62) = 3.16, *p* = .03, η_p_^2^ = .13 . To further explore this interaction, we looked at the four conditions separately. In both the CF and Ctrl categories, we found no significant changes. In the FO category however, there was a significant effect of time: F (1,12) = 4.931, *p* = .05, η_p_^2^ = .29. Mean scores dropped from 2.62 to 1.62, indicating that participants in this category lowered their thermostats during the course of the intervention. In the CO category, another significant effect of time was found: *F* (1,17) = 6.729, *p* = .02, η_p_^2^ = .28. Contrary to our expectations, mean scores in this category went up, from 2.60 to 3.20, indicating that participants in this group actually turned up their thermostat. Since these groups are very small, we must be cautious not to over-interpret these findings.

For lowering the thermostat when temperature in the office was too high there was an marginal effect of Time, *F*(1,78) = 2.85, *p* < .10, showing a tendency to quicker adjustment of thermostat setting after the intervention.

For closing the door when the central heating was on we again found an effect of time, *F*(1, 83) = 5.40, *p* < .03, η_p_^2^ = .067, and no more.

For closing the window when the central heating was on there were no effects of time, nor the interaction of time and intervention.

### Environmental Concern

To measure whether our intervention affected participants’ environmental concern we performed a MANOVA with experimental category as the independent variable and the five environmental concern items as the dependent variables. It is important to note that these items were only measured after the intervention had taken place (Table 2). What is notable is the favorable mean score on the second item, indicating that at the end of the intervention there was a general positive attitude towards energy conservation among our sample. At the same time we observe an unfavorable mean score on the fourth item, indicating that people did not experience much social pressure to save energy.

**Table 2:** Mean scores and standard deviations on environmental concern (post-intervention only)

Item	M	SD
‘I find it easy to conserve energy in my office’	2.41.	1.02
‘I find it important to conserve energy in my office’	1.90	.97
I feel guilty when I do not conserve energy in my office’	2.92	1.22
‘Colleagues address me when I do not conserve energy in my office’	3.96	1.15
‘I strongly intend to conserve energy in my office’	2.20	.98

Scores range from 1 (*agree strongly*) to 5 (*disagree strongly*)

Results show no effect of our intervention on any of the items, *F*(15,297) = 1.32, n.s. Number of persons per office, the covariate, showed a significant effect (F(5, 90) = 2.35, *p* < .05. A closer look at the individual items strongly suggests that the general effect was caused by the item ‘Colleagues address me when I do not conserve energy in my office.’ The low score on this item differs for different numbers of persons per office (*F* (4, 94) = 3.90, *p* < .01. Some normative pressure is experienced in offices with three or more persons (*M* = 3.7), and virtually no such influence in offices occupied by one or two persons (*M* = 4.6).

## Discussion

We tested the effects of an intervention containing commitment and feedback aimed to reduce energy use in an office environment. The intervention was based on state-of-the art scientific insights and carefully implemented. We were also extremely careful in our measurements of energy behavior and the delivery of feedback.

None of our three hypotheses was actually confirmed. We did not find the hypothesized effects of commitment (CO) or feedback (FO) or their combination (CF), compared to the control category (Ctrl). However, at the end of the program, for five of the behaviors (switching off lights, switching the computer to standby mode, and to sleep mode, lowering thermostats, and closing the door) we found a trend towards energy saving, although this was found in the entire sample and not, as hypothesized, only in the experimental treatment categories. So clearly we cannot attribute these behavior changes to our intervention. We found some effect of our feedback treatment, but this was limited to lowering the thermostat. In addition we found signs of the influence of the immediate social context: colleagues with whom an office was shared were apparently influential for switching off lights and switching off computers. In the environmental concern measure the effect of immediate colleagues stimulating energy saving was also found.

It seems that simply hearing about and engaging in an energy saving program was enough for employees to make (small) behavior changes. On the other hand, while a steady stream of research shows commitment to be an effective behavior change technique (Lokhorst *et al.*^2013^), especially when combined with feedback (DeLeon and Fuqua 1995), we were not able to replicate these findings in our study. This does not lead us to the conclusion that commitment and feedback are ineffective techniques. If we take into account that psychological studies usually have modest power (Cohen 1962), it should come as no surprise that some studies obtain non-significant results. This is by no means a sign of the effect not being reliable (Schimmack 2012).

However, it does give us room to reflect on what happened in this study. We need to take a closer look at our intervention and its implementation to understand what we could have done differently that would have produced a better result. By doing so, we can learn under what conditions commitment-making and feedback can be effective One very apparent issue regarding the commitment manipulation is that roughly one-third of the participants who were asked to do so agreed to make a commitment, meaning that more than two-thirds refused. These are disappointing numbers, giving rise to the questions why commitment was so unattractive here, and what can be done to get people to make commitments.

One reason why people were reluctant to make a commitment could be that we told them their names would appear on an internal memo, something they would rather avoid. We used the publication of the names deliberately, as it has been suggested that commitments should be made in public in order to make them effective (Cialdini 2001). While this may very well be the case, it might also scare people away from commitment making, rendering it less effective overall. It is likely that organizational culture plays a role here: whether people feel they will be rewarded for ‘putting themselves out there,’ or whether they expect indifference, disapproval, or even punishment. The unfavorable mean score on the item ‘Colleagues address me when I do not conserve energy in my office’ (Table 2) indicates that in this specific environment, energy saving was not seen as the norm.

Research by Handgraaf *et al.* (2013) on how different incentives might spur electricity savings in companies, showed that people are very sensitive towards public social rewards, even more so than towards financial rewards. It could be that here, people did not expect a reward and that this made commitment less attractive.

So how do we get people to commit? One answer to the problem of getting to make commitments is to ensure that the organizational culture rewards commitment. This can be done by preparing people for the intervention and by inviting them to be part of the intervention from the start. Such a participatory approach creates ownership (Israel *et al.*^2001^). If people feel they are part of the program, they are likely to see the programs’ goals as more suited to their own outlook; that is, as expressing their own interests and values (Sheldon and Elliot 1999). Such concordance makes the intervention more attractive and eventually more successful (Unsworth *et al.*^2013^).

A second option is to use a social norms approach. A plethora of research shows that people are sensitive to social norms, that is they are likely to model their behavior after that of others (Cialdini 2001; Nolan *et al.*^2008^). These findings can easily be applied in a commitment intervention: rather than simply asking people to put down their name, they could be shown a list of names of people who have already committed and asked to add their name. Even such a slight change will likely increase the number of people willing to make a commitment. Such a procedure might also involve the cooperation of role models, people with a strong influence on the organization. For example, the public commitment of the Mayor of Leiden to behave as energy efficiently as possible in his own office might have had a significant impact on participation in our program at the City Hall.

Another feature of this study that likely accounted for the lack of effect of commitment- making. After having made their initial commitment participants were never reminded of having done so. In order to be effective it might be necessary to keep the commitment salient (Lokhorst *et al.*^2013^) by providing participants with cues to remind them of their earlier decision. Granted, the continuous feedback in the CF category could be seen as such a cue, but, judging from results, probably not one strong enough to stimulate the energy saving goal. It is probable that for commitment-making to be successful we need to look at it as a *process* rather than a one-time intervention; the commitment needs to be reinforced throughout several steps in the program.

This process model is exemplified in Bamberg’s (2013) stage model of self-regulated behavioral change, in which people move through different stages of change. In the first pre-decisional stage, people are faced with different goals of which they have to choose one, and formulate it into a goal intention, such as ‘I intend to save energy.’ Since there are several behavioral strategies to accomplish such a goal, in the next pre-action phase, people need to formulate a behavioral intention that specifies how they will accomplish their goal. Such an intention could be ‘In order to save energy, I intend to switch off the lights when I leave my office.’ To go to the next action stage, people finally need to set an implementation intention that takes into account the context in which the behavior takes place. Such an implementation intention would be ‘When I leave my office to go to lunch at 12 PM, I will switch off my lights.’ Looking back on our study, our commitment manipulation most closely resembled a goal intention. If we look at commitment making as a process, then we probably should have followed up that goal intention with behavioral intentions and implementation intentions to help people adhere to their commitment.

A few more practical limitations of the current study might have hindered the intervention’s potential. Firstly the weather conditions changed during the course of the intervention: winter set in, and this probably affected energy use. Ideally, for an experiment such as this, you would want weather conditions remain the same across all phases of the study. This is, of course, impossible. With the outside temperatures decreasing, people tend to start using more energy and this may have impacted the effects of our intervention. We tried to account for changing weather conditions by selecting behaviors that we expect to be unaffected by seasonal fluctuations (Table 1). Nevertheless, it is a notable limitation. Second, while some employees had their own personal office, others shared with up to eight co-workers. It is probable that sharing an office can greatly influence the extent to which employees can and will change their behavior, for better or for worse. Our results suggest that, at least for some behaviors, the presence of co-workers was beneficial for energy saving. But sharing an office might also mean people perceive environmental behaviors as being not entirely under their own control, discouraging pro-environmental change. This is certainly an area for further research. Third, our study clearly lacks statistical power. Our sample size was limited, with an uneven distribution across conditions. This is an important impediment to our study as it lowers the probability of detecting an effect. Finally, in this study, we were unable to measure behavior directly but had to rely on participants’ self-reported behavior. While this approach is not uncommon in this type of research, it is also not without its drawbacks. Participants’ answers may for instance be subject to social desirability bias, or they may have simply forgotten their exact behavior of the past weeks. These limitations should be taken into account when evaluating our study.

Nevertheless, despite the lack of effects reported here, we still advocate commitment-making combined with feedback as a potentially useful intervention. Our research demonstrates potential pitfalls of such an approach and we have proposed several ways of dealing with them. At the outset commitment-making has to be made attractive for employees by engaging them in the process and by showing them that they are joining others who have committed as well. Second, the commitment needs to be reinforced through several steps in the program. This can be integrated with the feedback delivery, for instance by reminding participants of their commitment each time they receive feedback, and helping them in further specifying their commitment as they move through different stages of change.

The lessons learned here about how to design and implement a successful behavior change program are not exclusive to energy saving. The literature we draw upon is about pro-environmental behavior in general (cf. Handgraaf *et al.*^2013^). We do not regard the generic character of our message as an impediment but rather as a strength of our analysis and believe that a carefully introduced, well developed and implemented commitment intervention can be successful across a wide range of pro-environmental behaviors (Lokhorst *et al.*^2013^).

In conclusion, this study shows that commitment-making combined with feedback is no ‘one size fits all’ solution to energy use problems. It can be effective, but only when certain conditions have been met. We have tried to outline these conditions. We propose approaching commitment-making from a process perspective, whereby we first determine what is needed to persuade people to make a commitment, and then design our interventions so that people are enabled to transform their commitment into behavior change. With this paper we aim to contribute to the development of effective and theory- and evidence-based interventions.

## Appendix

**Table 3:** Distribution of response rates across conditions

	1st Questionnaire		Commitment		2nd Questionnaire		3rd Questionnaire	
Condition	Handed Out	Completed	Committed	Not committed	Handed out	Completed	Handed out	Completed
CF	53	45	18	27	54	32	45	40
CO	39	35	10	25			35	29
FO	25	24					24	20
Ctrl	29	26					26	19
Total	`146	130			54	32	130	108

*CF* commitment and feedback, *CO* commitment only, *FO* feedback only, and *Ctrl* control
